# Supplementary material for: Multitimescale Computation by Astrocytes
Source: bioRxiv. 2026 Jan 5:2026.01.05.697758. Preprint. [Version 1] doi: 10.64898/2026.01.05.697758 (PMC12803167; doi:10.64898/2026.01.05.697758)
Supplement: Supplementary file 1 [file NIHPP2026.01.05.697758v1-supplement-1.pdf]

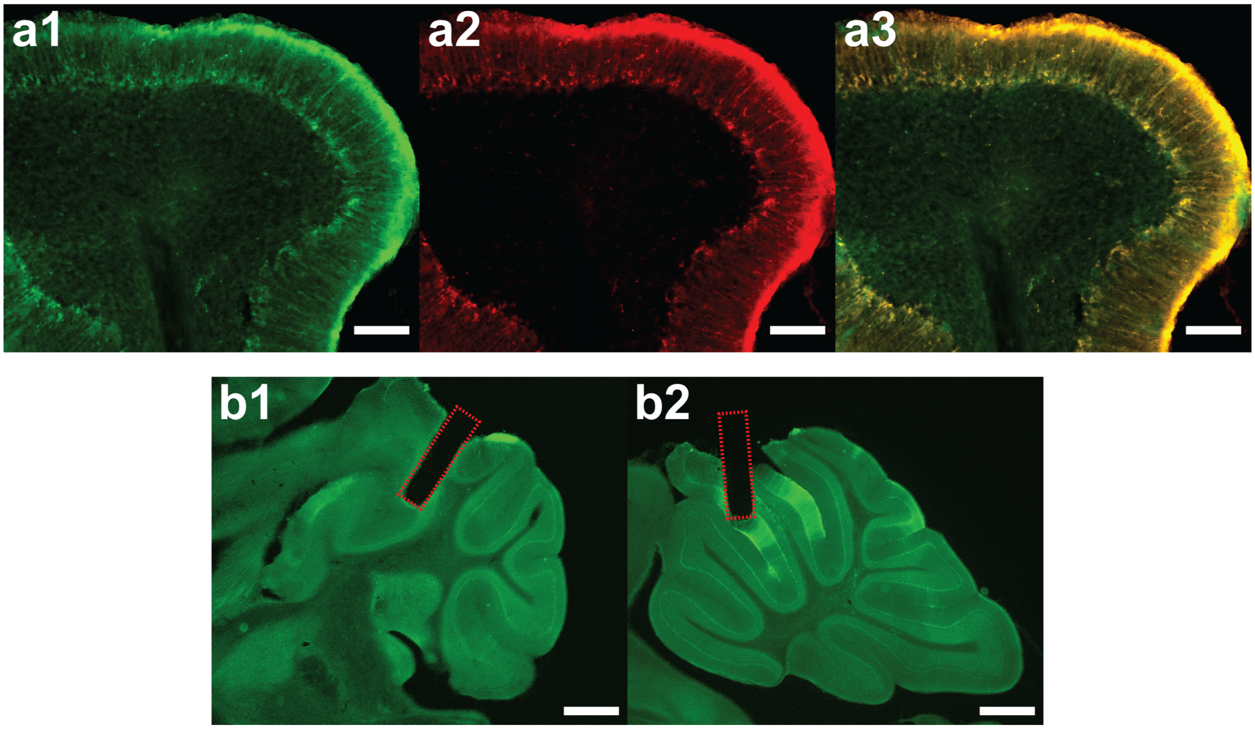

Supplemental Figure 1: Validation of Bergmann Glia Expression and Fiber Placement

(a1) GCaMP6f immunostaining (green) showing expression in Bergmann glia. Scale bar: 100 µm.
(a2) S100β astrocyte marker immunostaining (red). Scale bar: 100 µm.
(a3) Colocalization of GCaMP6f and S100β, confirming astrocyte-specific expression. Scale bar: 100 µm.
(b1) Fiber photometry cannula placement in left and right simplex. Scale bar: 1000 µm.
(b2) Fiber photometry cannula placement in midline lobule III. Scale bar: 1000 µm.

bioRxiv preprint doi: https://doi.org/10.64898/2026.01.05.697758; this version posted January 5, 2026. The copyright holder for this preprint (which was not certified by peer review) is the author/funder, who has granted bioRxiv a license to display the preprint in perpetuity. It is made available under aCC-BY 4.0 International license.

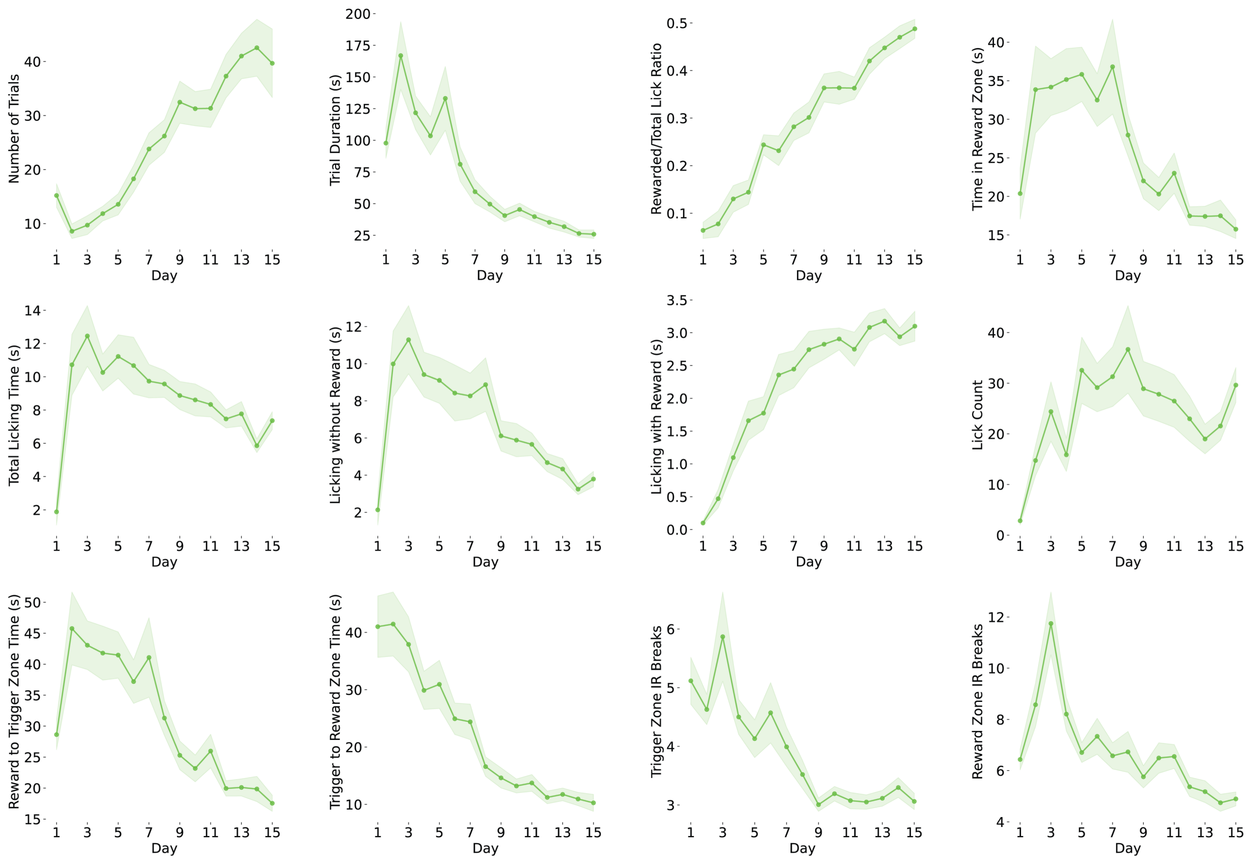

Supplemental Figure 2: Behavioral Learning Across Training

Training-dependent improvements (Days 1-15) in trial number, trial duration, rewarded/total lick ratio, reward zone time, total licking time, licking with/without reward, licking counts, trigger zone to reward zone time, reward zone to trigger zone time, and trigger zone/reward zone IR breaks.

bioRxiv preprint doi: https://doi.org/10.64898/2026.01.05.697758; this version posted January 5, 2026. The copyright holder for this preprint (which was not certified by peer review) is the author/funder, who has granted bioRxiv a license to display the preprint in perpetuity. It is made available under aCC-BY 4.0 International license.

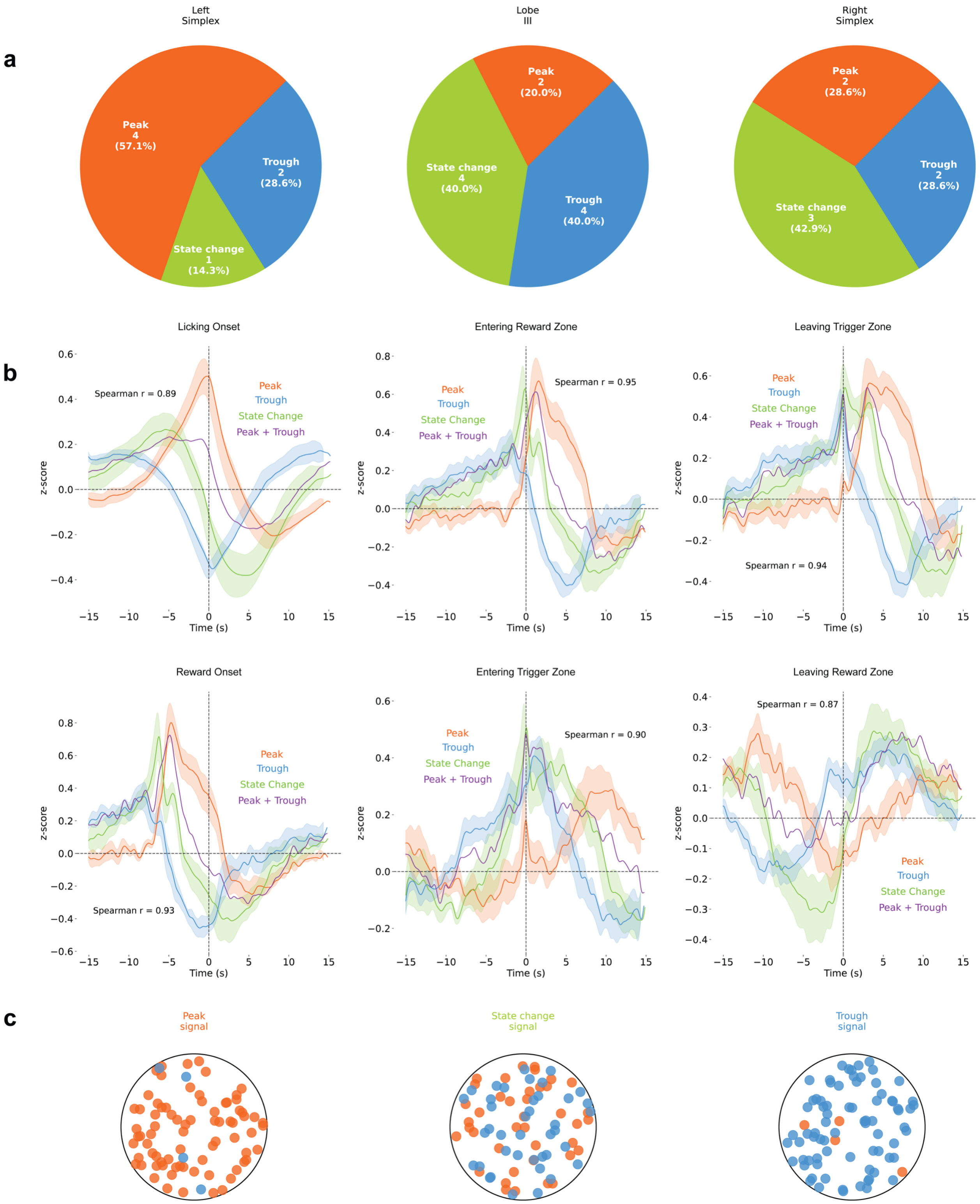

Supplemental Figure 3: Spatial Distribution of Slow Signal Types Across Cerebellar Regions

(a) Signal type distribution across left simplex, lobule III, and right simplex shows no clear anatomical bias.

bioRxiv preprint doi: https://doi.org/10.64898/2026.01.05.697758; this version posted January 5, 2026. The copyright holder for this preprint (which was not certified by peer review) is the author/funder, who has granted bioRxiv a license to display the preprint in perpetuity. It is made available under aCC-BY 4.0 International license.

(b) Sum of peak and trough signals (z-scored ΔF/F) approximates state-change dynamics (Spearman r = 0.87-0.95), suggesting population integration.
(c) Schematic of heterogeneous signal types within photometry field of view.

bioRxiv preprint doi: https://doi.org/10.64898/2026.01.05.697758; this version posted January 5, 2026. The copyright holder for this preprint (which was not certified by peer review) is the author/funder, who has granted bioRxiv a license to display the preprint in perpetuity. It is made available under aCC-BY 4.0 International license.

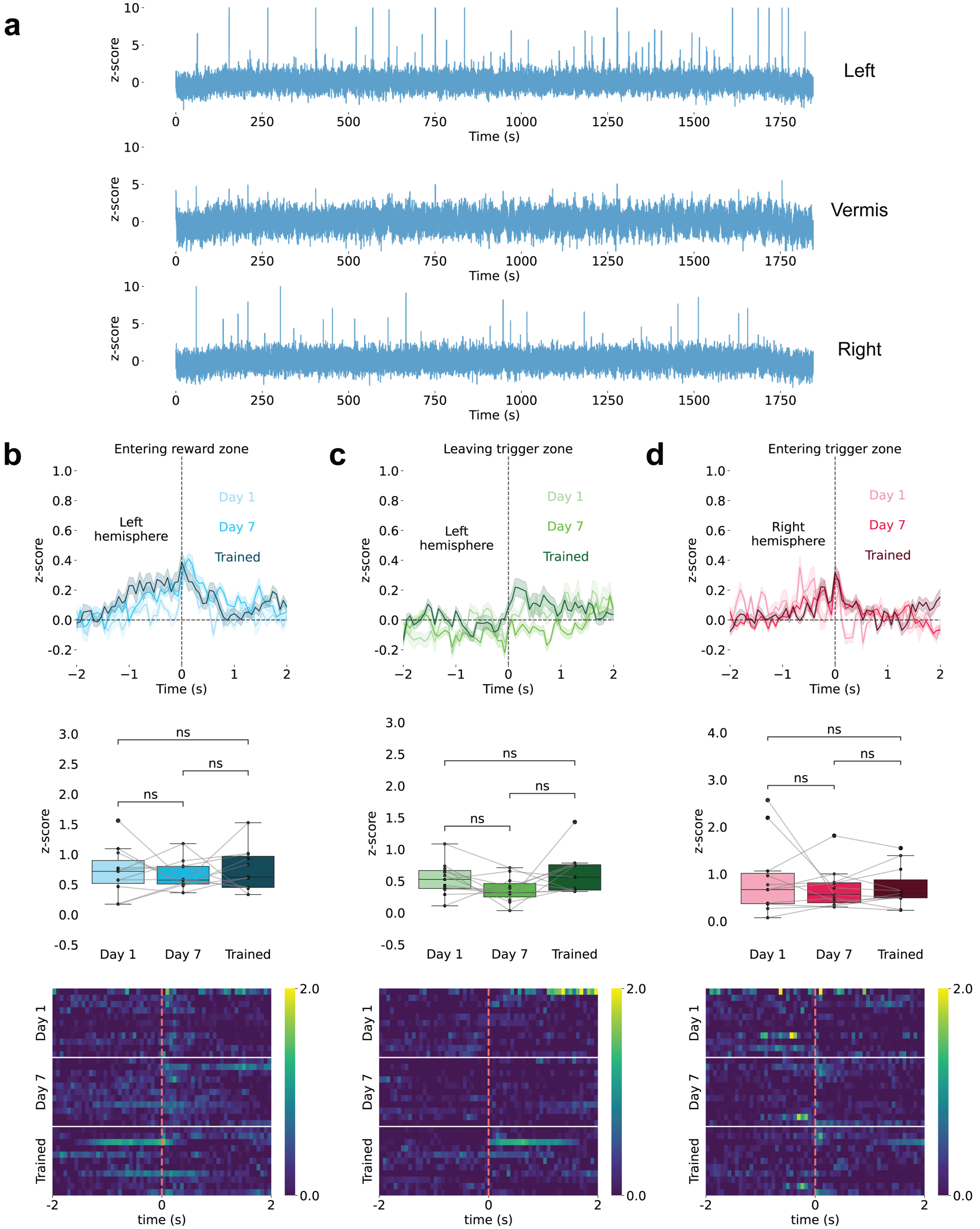

Supplemental Figure 4: Raw Traces Showing Fast Transients Across Recording Regions

(a) Fast transients (z-scored ΔF/F) in lateral simplex but not midline lobule III.

bioRxiv preprint doi: https://doi.org/10.64898/2026.01.05.697758; this version posted January 5, 2026. The copyright holder for this preprint (which was not certified by peer review) is the author/funder, who has granted bioRxiv a license to display the preprint in perpetuity. It is made available under aCC-BY 4.0 International license.

(b-d) Lobule III shows no significant fast responses (z-scored ΔF/F) to **(b)** entering reward zone, **(c)** leaving trigger zone, or **(d)** entering trigger zone across training (n=11 sites, 11 animals).

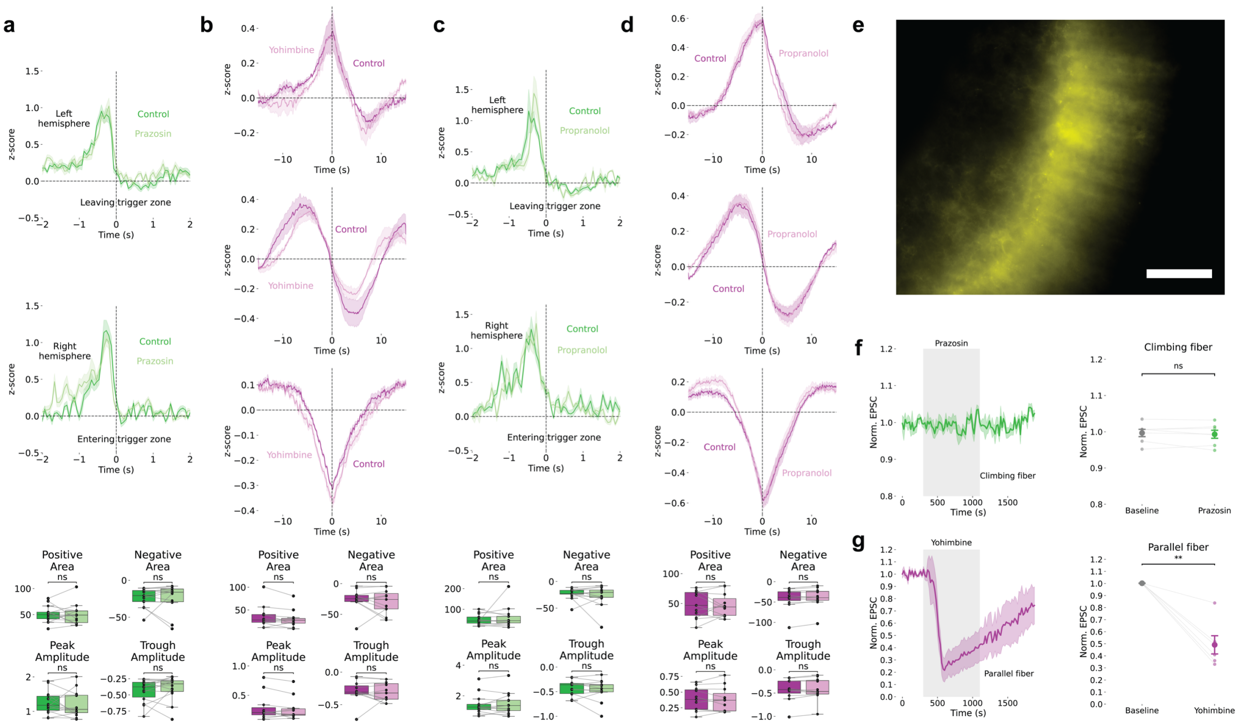

Supplemental Figure 5: Pharmacological Specificity of Adrenergic Receptor Effects

(a) Prazosin (α1-AR antagonist) does not affect fast transients (z-scored ΔF/F).
(b) Yohimbine (α2-AR antagonist) does not affect slow signals (z-scored ΔF/F).
(c-d) Propranolol (β-AR antagonist) does not affect slow or fast signals (z-scored ΔF/F).
(e) PMCA expression in Bergmann glia. Scale bar: 50 μm.
(f) Prazosin does not affect CF-evoked EPSCs.
(g) Yohimbine reduces PF-evoked EPSCs.

bioRxiv preprint doi: https://doi.org/10.64898/2026.01.05.697758; this version posted January 5, 2026. The copyright holder for this preprint (which was not certified by peer review) is the author/funder, who has granted bioRxiv a license to display the preprint in perpetuity. It is made available under aCC-BY 4.0 International license.

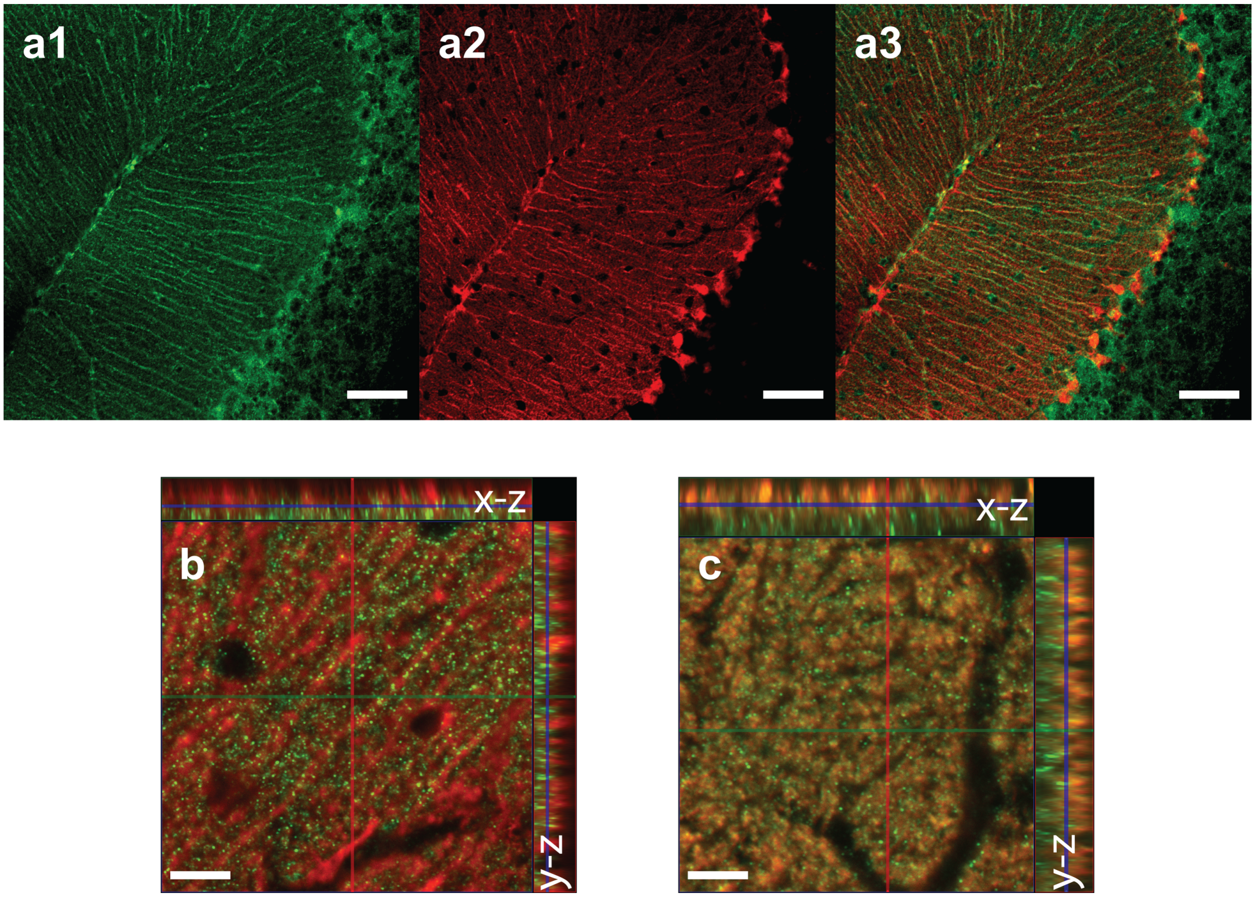

Supplemental Figure 6: Anatomical Localization of Adrenergic Receptors

(a1) α2-AR immunostaining showing expression in cerebellar cortex Bergmann glia molecular layer. Scale bar: 50 µm.

(a2) S100β astrocyte marker immunostaining. Scale bar: 50 µm.

(a3) Colocalization of α2-AR and S100β. Scale bar: 50 µm.

(b) Colocalization of α1-AR and S100β astrocyte marker. Scale bar: 20 µm.

(c) Colocalization of α1-AR and VGLUT1-positive parallel fiber terminals. Scale bar: 20 µm.

bioRxiv preprint doi: https://doi.org/10.64898/2026.01.05.697758; this version posted January 5, 2026. The copyright holder for this preprint (which was not certified by peer review) is the author/funder, who has granted bioRxiv a license to display the preprint in perpetuity. It is made available under aCC-BY 4.0 International license.

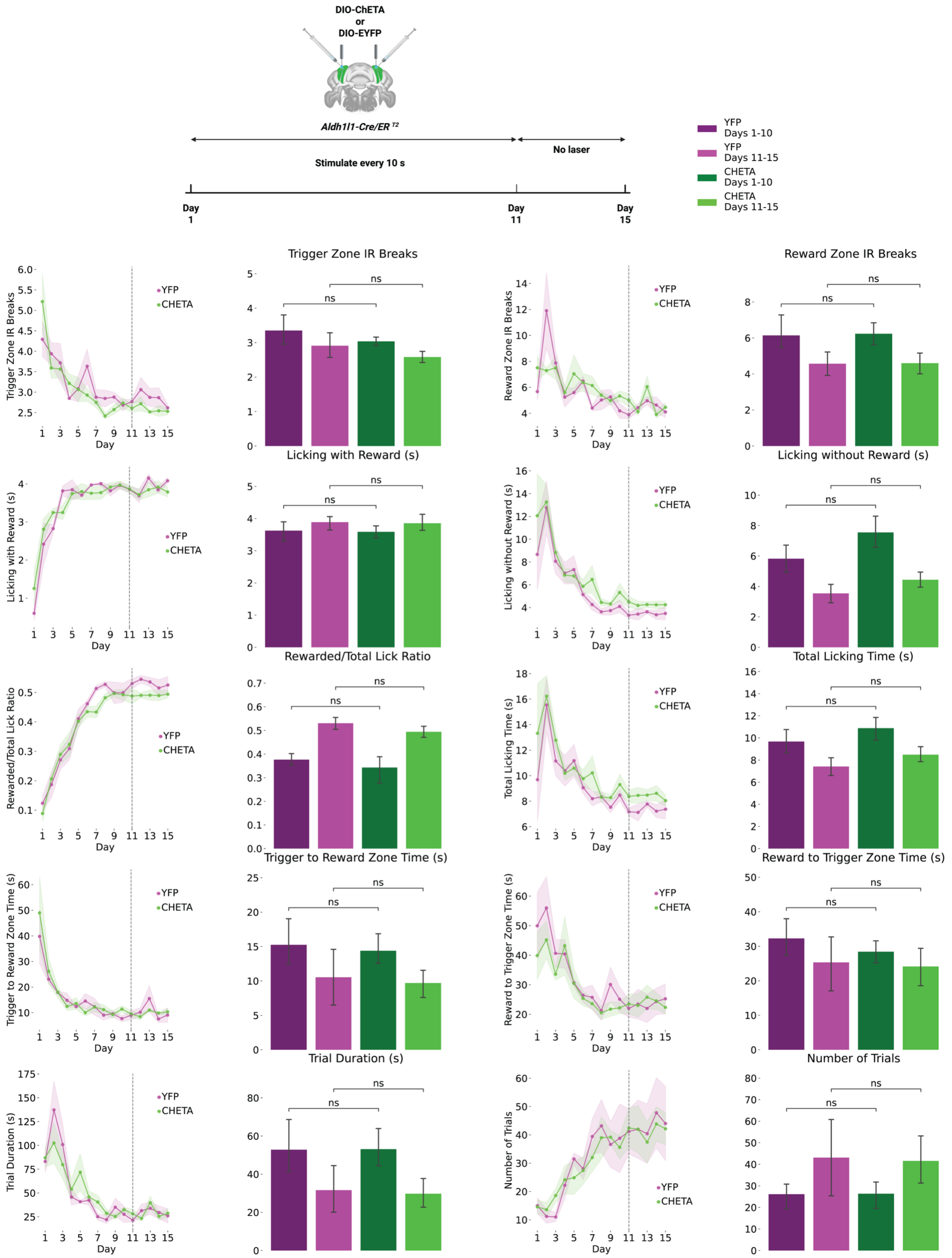

bioRxiv preprint doi: https://doi.org/10.64898/2026.01.05.697758; this version posted January 5, 2026. The copyright holder for this preprint (which was not certified by peer review) is the author/funder, who has granted bioRxiv a license to display the preprint in perpetuity. It is made available under aCC-BY 4.0 International license.

Supplemental Figure 7: Task-Independent Effects of Optogenetic Fast Signal Disruption

ChETA stimulation delivered at regular intervals (every 10 seconds independent of behavior) during Days 1-10 (n=7 ChETA, n=5 YFP control). Task-independent stimulation produces no behavioral changes compared to EYFP control, demonstrating that fast BG signal disruption requires temporal coupling to task-relevant cues (as shown in Figure 3) and is not due to nonspecific optogenetic activation.
